# Design of the Arizona CoVHORT: A Population-Based COVID-19 Cohort

**DOI:** 10.3389/fpubh.2021.620060

**Published:** 2021-02-10

**Authors:** Collin J. Catalfamo, Kelly M. Heslin, Alexandra Shilen, Sana M. Khan, Josh R. Hunsaker, Erika Austhof, Leila Barraza, Felina M. Cordova-Marks, Leslie V. Farland, Pamela Garcia-Filion, Joshua Hoskinson, Megan Jehn, Lindsay N. Kohler, Karen Lutrick, Robin B. Harris, Zhao Chen, Yann C. Klimentidis, Melanie L. Bell, Kacey C. Ernst, Elizabeth T. Jacobs, Kristen Pogreba-Brown

**Affiliations:** ^1^Department of Epidemiology and Biostatistics, Mel and Enid Zuckerman College of Public Health, The University of Arizona, Tucson, AZ, United States; ^2^Department of Community, Environment and Policy, Mel and Enid Zuckerman College of Public Health, The University of Arizona, Tucson, AZ, United States; ^3^University of Arizona Cancer Center, Tucson, AZ, United States; ^4^Department of Biomedical Informatics, College of Medicine–Phoenix, The University of Arizona, Phoenix, AZ, United States; ^5^School of Human Evolution and Social Change, Arizona State University, Tempe, AZ, United States; ^6^Department of Health Promotion Sciences, Mel and Enid Zuckerman College of Public Health, The University of Arizona, Tucson, AZ, United States; ^7^Department of Family and Community Medicine, College of Medicine–Tucson, The University of Arizona, Tucson, AZ, United States

**Keywords:** COVID-19, SARS-CoV-2, cohort study [or longitudinal study], epidemiology, long-term follow up

## Abstract

This study is a prospective, population-based cohort of individuals with a history of SARS-CoV-2 infection and those without past infection through multiple recruitment sources. The main study goal is to track health status over time, within the diverse populations of Arizona and to identify the long-term consequences of COVID-19 on health and well-being. A total of 2,881 study participants (16.2% with a confirmed SARS-CoV-2 infection) have been enrolled as of December 22, 2020, with a target enrollment of 10,000 participants and a planned follow-up of at least 2 years. This manuscript describes a scalable study design that utilizes a wide range of recruitment sources, leveraging electronic data collection to capture and link longitudinal participant data on the current and emerging issues associated with the COVID-19 pandemic. The cohort is built within a collaborative infrastructure that includes new and established partnerships with multiple stakeholders, including the state's public universities, local health departments, tribes, and tribal organizations. Challenges remain for ensuring recruitment of diverse participants and participant retention, although the electronic data management system and timing of participant contact can help to mitigate these problems.

## Introduction

SARS-CoV-2, and its associated disease state COVID-19, have been increasing in incidence globally since the first detailed report emerged in February 2020 (1). We are currently in an unprecedented public health response aimed at controlling disease spread (2), and there is an urgent need to capture and evaluate key information about pathogen transmissibility and risk factors associated with severe disease (3). It is equally important to understand symptom duration and the short and long-term health sequelae of SARS-CoV-2 infection. Management and prevention strategies can be developed and applied for patients to mitigate the adverse impact of COVID-19 on their overall health and well-being.

Early reports of COVID-19 epidemiology identified increasing age and several comorbidities as major risk factors for the development of a severe course of disease (4). Later, more refined data clarified that specific cardiometabolic conditions, including hypertension, diabetes, and cerebrovascular disease, increase the risk of adverse outcomes for COVID-19 patients (5). Reports of the infection in patients from Wuhan, China initially indicated that COVID-19 was largely a respiratory illness, with the majority of patients presenting with shortness of breath, dry cough, and, among the severely ill, Acute Respiratory Distress Syndrome (ARDS) (6). Subsequent studies revealed the involvement of multiple organ systems, including cardiovascular, neurological, and gastrointestinal. Cardiovascular manifestations include unspecified arrhythmia (1, 7), acute cardiac injury (1, 7), cardiomyopathy (6), heart failure (7), and thrombotic complications (7, 8). Additionally, the risk of acute ischemic stroke was found to be higher among COVID-19 patients as compared to patients with influenza (9). Neurological complications and cognitive impairment have been observed in up to 69% of COVID-19 patients including delirium, anosmia, headache, and dizziness, as well as the more severe sequelae of Guillain-Barre Syndrome and encephalitis (10–12). Gastrointestinal (GI) symptoms, initially comparatively rare among patients in China (13), have been more prevalent in patients in other countries with up to 61.3% of patients infected with SARS-CoV-2 presenting with at least one GI symptom in a multi hospital study conducted in the United States (14). However, due to the novelty of the virus and the lack of available treatment options, there is a paucity of knowledge about which chronic health conditions are triggered or aggravated by COVID-19 disease.

In addition to the growing need for data on the conditions that arise from infection with SARS-CoV-2, the duration of symptomatology and heterogeneity in symptom profiles are currently unknown. Among a group of 143 hospitalized COVID-19 patients in Italy, 87.4% continue to experience at least one symptom for up to 60 days post-recovery, of which lasting fatigue and dyspnea were the most prevalent (15). The majority of these patients report significant reductions in their quality of life (QoL) compared to the time prior to their illness (15). A growing number of social media users report their illness as lasting >60 days post-recovery from the acute phase; however, these reports are anecdotal, unconfirmed, and require systematic analysis within the context of a large, prospective epidemiological investigation.

We know from past outbreaks of related coronaviruses that there is a potential for chronic health outcomes following the acute infection. Cohorts established following the 2002–2003 SARS epidemic have identified a range of health conditions among survivors of the infection. Survivors have reported a reduced QoL and persistent fatigue/malaise for up to 1 year after recovery from the acute phase (16). These cohort studies following the SARS epidemic have also reported SARS survivors experiencing an increased susceptibility to lung infection, altered metabolisms, and cardiovascular abnormalities for more than a decade after recovery (17), and lung and bone injury persisting 15 years post-recovery (18). The combination of prolonged sequelae associated with previous coronavirus outbreaks and early reports of sequelae among COVID-19 patients suggests that COVID-19 illness could precipitate a new wave of prolonged chronic diseases accompanying an acute infection symptomology. These past studies underscore the urgency for the rapid development of a rigorous and robust longitudinal study to compare outcomes in patients infected with SARS-CoV-2 to uninfected individuals.

In addition to research gaps related to comorbidities and sequelae associated with COVID-19, the identification of factors that may precipitate a more severe course of disease is urgently needed. A growing number of reports detailing severe COVID-19 cases in young adults with no known underlying conditions underscores the need for carefully designed prospective epidemiology studies to determine why some individuals progress to severe forms of disease and others do not. Further, the short- and long-term health impacts of SARS-CoV-2 infection in asymptomatic individuals is unknown. In one report, abnormal radiological findings of the lung were identified in 66.7% (14/21) of asymptomatic individuals (19), but the comparatively small sample size and short duration of this study evinces the need for a larger epidemiological study with longer follow-up.

In this manuscript, we describe the development of a prospective, population-based cohort of COVID-19 positive participants and uninfected population-based participants designed to (1) determine the contribution of putative risk factors and extant comorbidities to COVID-19 disease severity, (2) identify chronic health conditions that arise following COVID-19 disease, (3) and examine the relationship between disease severity and chronic health outcomes. This Arizona COVID-19 Cohort, dubbed “CoVHORT,” will enable us to determine the prevalence of patient-reported health outcomes throughout the acute phase of the illness in baseline assessments, and incident outcomes during the recovery phase. Our prospective approach enables us to identify risk factors or exposures that are implicated in the development of post-COVID-19 health outcomes, including new onset of chronic conditions, exacerbation of existing conditions, or persistence of COVID-19 symptoms. Our inclusive recruitment strategies allow us to reach a diverse population of Arizona residents, increasing the generalizability of our findings to rapidly inform prevention and treatment strategies. A population-based prospective cohort of patients with and without COVID-19 diagnosis is an ideal study design to collectively investigate the COVID-19 disease course including risk factors, disease progression, resolution, and chronic outcomes of infection.

## Methods and Analysis

### Survey Instruments

The baseline survey of the CoVHORT is a 95-item questionnaire using multiple query formats, including multiple choice, multi-select, and free-response text fields. To prepare the survey instruments, we first conducted a review of previously published literature in order to develop survey items that encompass all reported symptoms of the infection. Additional questions, standard to case investigations and studies of infectious diseases, include duration of symptoms and potential exposure routes. Due to the dearth of information regarding household transmission, and particularly transmission from children to adults, the survey includes items to characterize participant household structure and to track spread of infection among household members. In addition to the peer-reviewed literature, public reports of chronic, persistent symptoms were included based on review of symptoms reported by COVID-19 patients using the hashtag “#longcovid” on social media websites. The inclusion of these symptoms allows for a rigorous approach to confirm whether these symptoms are more common in those who have been infected with SARS-CoV-2 as compared to uninfected individuals. We continue to review the COVID-19 literature weekly so that we can update our questionnaires to reflect new information as needed.

A multitude of social, individual, environmental and economic factors influence the acute phase of COVID-19, and these factors also play a significant role in the pathology of other chronic diseases. For this reason, the surveys were designed to capture domains that were impacted by the COVID-19 epidemic in Arizona, as well as information on the impact of COVID-19 on general well-being including financial hardship, risk perception, physical activity, sleep, pregnancy and maternal/fetal outcomes, and stress and emotional wellness (Tables 1, 2). The majority of the survey items were developed from the broad expertise of members of the Arizona CoVHORT research team, expanding on, and modifying previously validated scales.

**Table 1:** Topic areas included in administered surveys developed for the Arizona CoVHORT.

**Topic areas**	**Example questions**
**Acute illness**	
Duration	Since the start or your illness, are you feeling back to normal?
Exposures	In the 14 days before you started feeling ill, did you have close contact with someone who was positive for COVID-19?
Household transmission	How many people living in your home were confirmed or suspected to have had COVID-19 since your last survey?
Results of diagnostic testing	What were your COVID-19 test results?
Symptoms	Which symptoms did you experience during your illness?
Severity	Were you hospitalized for this illness?
**Chronic illness**	
Changes to routine medical care	Have you or a family member missed routine or preventative health care since your last survey? This could include dentist appointments, physicals, eye exams, screening, or vaccination appointments for you or your children.
Development of chronic sequelae	Have you been newly diagnosed with any of the following conditions since your last completed survey? Were you diagnosed with this condition(s) before or after your COVID-19 diagnosis?
Exacerbation of pre-existing chronic health conditions	Since your last survey, have you experienced new complications, increases in severity, or changes in medication for any of the following conditions?
Persisting symptoms	Since your last completed survey have you noticed any of the following ongoing or new symptoms?
Financial hardship	How would you describe the money situation in your household right now?
Perceived risks and attitudes	In your opinion, how effective are the following actions for keeping you safe from COVID-19?
Physical activity	If you wear a wearable fitness tracker (e.g., Apple Watch, Fitbit, Garmin), have you noticed that your physical activity has changed compared to before the COVID-19 outbreak?
Pregnancy and reproduction	Have you tried to become pregnant or are you actively trying to become pregnant?
Perception of policy interventions	Should the government quarantine those who might have been exposed to COVID-19 to limit their contact with others?
Sleep quality	Since the start of the pandemic, how often have you had trouble sleeping? (This can be due to reasons such as trouble falling asleep, waking up early/in the middle of the night, trouble breathing/coughing/snoring, feeling too hot/cold, bad dreams, or pain).
Stress and emotional wellness	In the last month, how often have you been upset because of something that happened unexpectedly?

**Table 2:** Topic areas included in the Arizona CoVHORT surveys and their assessment frequencies over the course of the 1st year of follow-up.

**Topic areas**	**Baseline**	**3 months**	**6 months**	**9 months**	**1 year**	**“Off cycle”**
**Acute illness**						
Duration	∙	∙	∙	∙	∙	∙
Exposures	∙	∙	∙	∙	∙	
Household transmission	∙	∙	∙	∙	∙	
Results of diagnostic testing	∙	∙	∙	∙	∙	
Symptoms	∙	∙	∙	∙	∙	∙
Severity	∙	∙	∙	∙	∙	∙
**Chronic illness**						
Changes to routine medical care			∙		∙	
Development of chronic sequelae		∙	∙	∙	∙	∙
Exacerbation of pre-existing chronic health conditions		∙	∙	∙	∙	∙
Persisting symptoms	∙	∙	∙	∙	∙	∙
Financial hardship		∙		∙		
Perceived risks and attitudes		∙		∙		
Physical activity	∙		∙		∙	
Pregnancy and reproduction	∙	∙	∙	∙	∙	
Perception of policy interventions			∙		∙	
Sleep quality	∙		∙		∙	
Stress and emotional wellness		∙	∙	∙	∙	

#### Risk Factors for Infection and Severe Disease

At baseline, we ask information on behavioral factors currently hypothesized to influence risk of infection including: travel history (international and domestic), close contact with someone who tested positive for COVID-19, quarantine practices (among individuals with SARS-CoV-2 infection), living arrangements, household structure, household member illness history, participation in state-mandated closures (i.e., sheltering in place, working from home), employment-related exposures (i.e., essential worker; number of interactions with other people), employment location (e.g., working from home), volunteer-related exposure (e.g., fire department, school), personal COVID-19 mitigation efforts (e.g., mask wearing), and cigarette smoking/vaping/marijuana use. The questionnaire will further collect information on health and medical history that may contribute to risk of severe COVID-19 disease. Health and medical questions include information on height and weight to calculate body mass index (BMI), and pre-existing conditions. Individuals are asked general information about their ability to obtain regular treatment for controllable conditions including diabetes and high blood pressure to determine if challenges in chronic disease management may influence severity of COVID-19 illness. Information on medications known to lower immune function and whether the participant is actively on dialysis are also collected.

#### Chronic Disease History and Development

A baseline health history is taken. Among individuals with COVID-19 disease, participants are asked whether they had a history of clinician-diagnosed chronic disease prior to COVID-19 diagnosis. Among participants without a history of COVID-19, we ask about their history of clinician-diagnosed chronic disease prior to enrollment. On each follow-up questionnaire, all our participants are queried regarding changes in their health history and newly clinician-diagnosed conditions. The questionnaire asks specifically about cardiometabolic conditions (i.e., diabetes, pre-diabetes, gestational diabetes, hypertension, hypercholesterolemia, myocardial infarction, angina, congestive heart failure stroke, other cardiac/heart condition), respiratory conditions (i.e., asthma, valley fever, emphysema/chronic bronchitis, chronic obstructive pulmonary disease [COPD]), cancer history, influenza, thyroid disorders, liver disease, chronic kidney disease, gastrointestinal conditions (inflammatory bowel disease, ulcerative colitis, irritable bowel, acid reflux), mental health history (i.e., depression, anxiety), neurologic conditions (Parkinson's disease, lupus, multiple sclerosis), arthritis, and other (open response). Participants are also asked about changes in their condition including any new complications, increases in severity, or changes in the medication used.

#### Financial Hardship

Measures of financial hardship or deprivation assess whether individuals are excluded from minimally accepted ways of life in society due to a lack of resources (20). In contrast to income or poverty measures which infer exclusion from a lack of resources, financial hardship directly assesses the extent to which individuals or households lack goods, facilities, or services or are unable to engage in activities (21). There is consistent evidence that hardship is associated with psychological distress and common physiological disorders that are expected to be significant comorbidities with COVID-19 (22). We are utilizing a 6-item measure of financial hardship that includes assessments of personal, household, and medical needs that is currently being utilized in comparable COVID-19 projects (23). Both direct and indirect impacts of COVID-19 on financial hardship can be identified in this cohort.

#### Risk Perception

Contemporary assessments of risk perception focus on multiple dimensions such as affective (degree of concern and emotional attachment), deliberative (the probability of incident), and experience (consequence or impact) to determine the degree to which perception is likely to shape risk-oriented behavior (24). Recent studies have fielded survey instruments that assess that a tripartite factor structure, including all three dimensions, that best capture the degree of worry expressed by individuals about most health outcomes and the likelihood of taking protective measures (25). Our assessment of risk perceptions is pulled from the COVID-19 OBSSR Research Tool and is being utilized in comparable assessments (26, 27) to determine how perceptions of the risks and consequences of COVID-19 shape behaviors and outcomes.

#### Physical Activity and Sleep

Our participants are asked about their physical activity and sleep patterns prior to March 2020 and at time of the questionnaire. The questions on physical activity are modeled after the International Physical Activity Questionnaire (IPAQ) (28) and questionnaires used by the Women's Health Initiative (29) and ask about frequency and intensity over the past month. We also ask about information on average time to bed, time to rise, and sleep quality from participants to measure sleep duration and quality, in addition to whether their physical activity and sleep patterns have been affected by the COVID-19 pandemic.

#### Pregnancy and Maternal/Fetal Outcomes

Female, non-binary and trans male participants between 18 and 49 years of age are asked on each questionnaire whether they are currently pregnant, have become pregnant since their previous questionnaire, are actively trying to become pregnant, or are not trying to become pregnant. If a participant reports that they were pregnant but are no longer pregnant since their previous questionnaire, we collect information regarding the outcomes of their previous pregnancy including: result of their pregnancy (live birth, miscarriage, or termination), due dates, date of miscarriage or termination, date of delivery, and reasons for termination of pregnancy.

#### Stress

We are utilizing the perceived stress scale-10 (PSS-10) to ask our participants about their perceived stress in regard to COVID-19 over the course of follow-up. The 10-item validated PSS-10 assesses how unpredictable, uncontrollable, and overwhelming an individual may find their circumstances and has been shown to effectively capture stress over the previous 4–8 weeks in community-based samples with at least an 8th grade education (30). The impact of stress on the body is driven by the cognitively mediated responses to a stressful event, rather than the event itself. Thus, the PSS-10 is considered a better measure of relevant stress than objective measures of stressful events (30).

### Study Design

The Arizona CoVHORT is a population-based prospective cohort study in which 2,881 participants have been enrolled as of December 22, 2020. The enrollment goal is 10,000 Arizona residents that represent Arizona's geographic and demographic diversity across rural and urban areas. All Arizona residents are eligible to participate. Once enrolled, we determine their previous infection status by asking them to self-report clinical and/or serological results, history of a diagnosis, or symptoms consistent with COVID-19; SARS-CoV-2 infection status is reevaluated at each follow-up survey time point (see SARS-CoV-2 Infection Status and Participant Follow-up and Retention). All study procedures, including advertising, recruitment, consent, enrollment, and follow-up, are available in English and Spanish and conducted in accordance with approval by the University of Arizona Institutional Review Board (#2003521636A002) under the aegis of the University of Arizona Human Subjects Protection Program.

#### Eligibility Criteria

The eligibility criteria for participation are: (1) current resident of Arizona; (2) ability to complete a survey written in either English or Spanish; and (3) provide informed consent to participate. Participant who wish to participate, but are not current residents of Arizona, are excluded from the cohort. Other than current residence, we have no other exclusion criteria for participation. Individuals under 18 years old may participate in the study, provided that the parent or legal guardian gave informed consent and the minor provides assent to participate. Parent or legal guardians may provide survey data for household members that are younger than 14 years of age. Although all races and ethnicities are eligible for this study, we are not actively recruiting American Indian/Alaska Natives on or off tribal lands in Arizona. We are in the process of consulting with local tribes and tribal health organizations in Arizona to develop potential collaborations.

#### Recruitment

**Figure 1:**
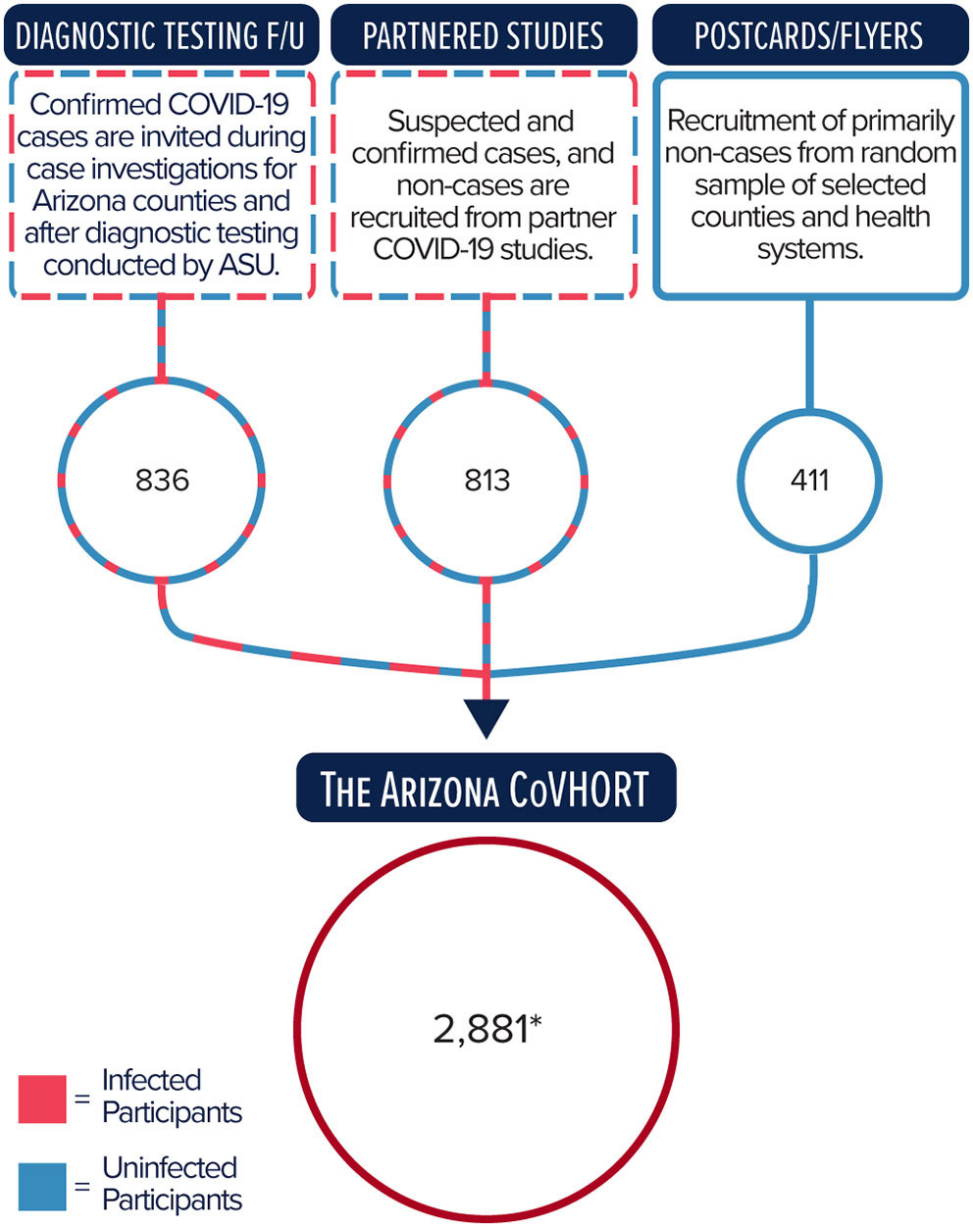
Primary recruitment methods and number of participants enrolled into the Arizona CoVHORT as of December 22, 2020. ^*^Total includes participants also recruited through other methods and by word of mouth.

Participants of the Arizona CoVHORT are recruited via three primary mechanisms, as illustrated in Figure 1. The first is composed of academic-public health department partnerships that provides diagnostic testing and conducts case investigations for SARS-CoV-2 positive individuals across Arizona. Prior to the COVID-19 outbreak, and in collaboration with local health departments, the Student Aid for Field Epidemiology Response (SAFER) program at the University of Arizona College of Public Health (31, 32) trained students to conduct case investigations for routine surveillance and outbreak response for various infectious diseases. In response to the COVID-19 pandemic, SAFER leveraged its existing infrastructure to train a large pool of undergraduate and graduate public health student volunteers to conduct case investigations for COVID-19 through established partnerships with the Arizona Department of Health Services and local health departments. Upon completion of each case investigation, cases are given the opportunity to provide their email address and automatically receive the electronic consent form for the Arizona CoVHORT study. Arizona State University's Biodesign Institute has also partnered with the Arizona Department of Health Services to provide free saliva diagnostic testing to underserved Arizona communities (https://biodesign.asu.edu/research/clinical-testing/testing). Individuals who participate in these testing opportunities who indicate interest in taking part in future research are also sent invitations to participate in the study. As of December 22, 2020, we recruit from six of 15 counties that encompass 90% of the state's population. These counties are a mix of rural and urban communities. To date, we have recruited 244 laboratory confirmed COVID-19 positive participants and 592 laboratory confirmed COVID-19 negative participants from this method, or 29.0% of our current total recruitment.

COVID-19 positive and negative participants will also be identified and enrolled through our established collaborations with several COVID-19-related research studies at the University of Arizona and Arizona State University (Figure 1). For example, following IRB approval and participant consent, participants in the State of Arizona COVID-19 Antibody Testing Initiative (https://covid19antibodytesting.arizona.edu/) who indicate interest in taking part in future research are sent invitations to CoVHORT in batches following the receipt of their results. This process is also applied to individuals who are current participants, or are ineligible or opted out of participating in the AZ HEROES study (https://azheroes.arizona.edu/). As of December 22, 2020, 813 of these participants (28.2% of our current total recruitment) have enrolled in the CoVHORT.

The final major recruitment channel is through a phased postcard mailing campaign to establish a population base of individuals who have not yet been infected with SARS-CoV-2 (Figure 1). The procedure consists of three rounds of mailed recruitment postcards to a simple random sample of 17,500 residences from each county selected. Phased mailings of recruitment postcards occur every 2 weeks to allow potential participants time to receive the material, review the consent materials, and choose whether or not to enroll in the study. This method enables us to maximize participation and minimize bias (33). We employ participant-provided information from baseline surveys to exclude addresses of those who have enrolled from the mailing list of each subsequent phase of the mailing campaign. Furthermore, each list is screened prior to each mailing to reduce the number of undeliverable postcards. We have completed the phased mailing campaign in Pima County, AZ and completed additional mailing campaigns in Pinal and Yuma County, AZ having contacted more than 51,000 households. We will next expand the postcard mailings to the other counties for which we are conducting case investigations, followed by the remaining Arizona counties. This method has resulted in the enrollment of 403 participants (14.0% of our current total enrollment).

In addition to direct invitations to individuals with laboratory confirmed infections identified through health department investigations, partnering COVID-19 study participants and testing sites, and the postcard mailing campaign, we have also created electronic and print recruitment materials. Recruitment flyers are in circulation within the Veteran's Affairs facilities and other healthcare systems in the greater Phoenix area. Future recruitment efforts will be focused on onboarding additional counties to the CoVHORT through case investigations with other jurisdictions and partnering with additional COVID-19 research studies in Arizona. Postcard mailings will also be directed to each of these counties to continue to expand the cohort. These efforts will be informed by our initial campaigns and tailored as necessary for maximum return and community reach.

#### SARS-CoV-2 Infection Status

**Figure 2:**
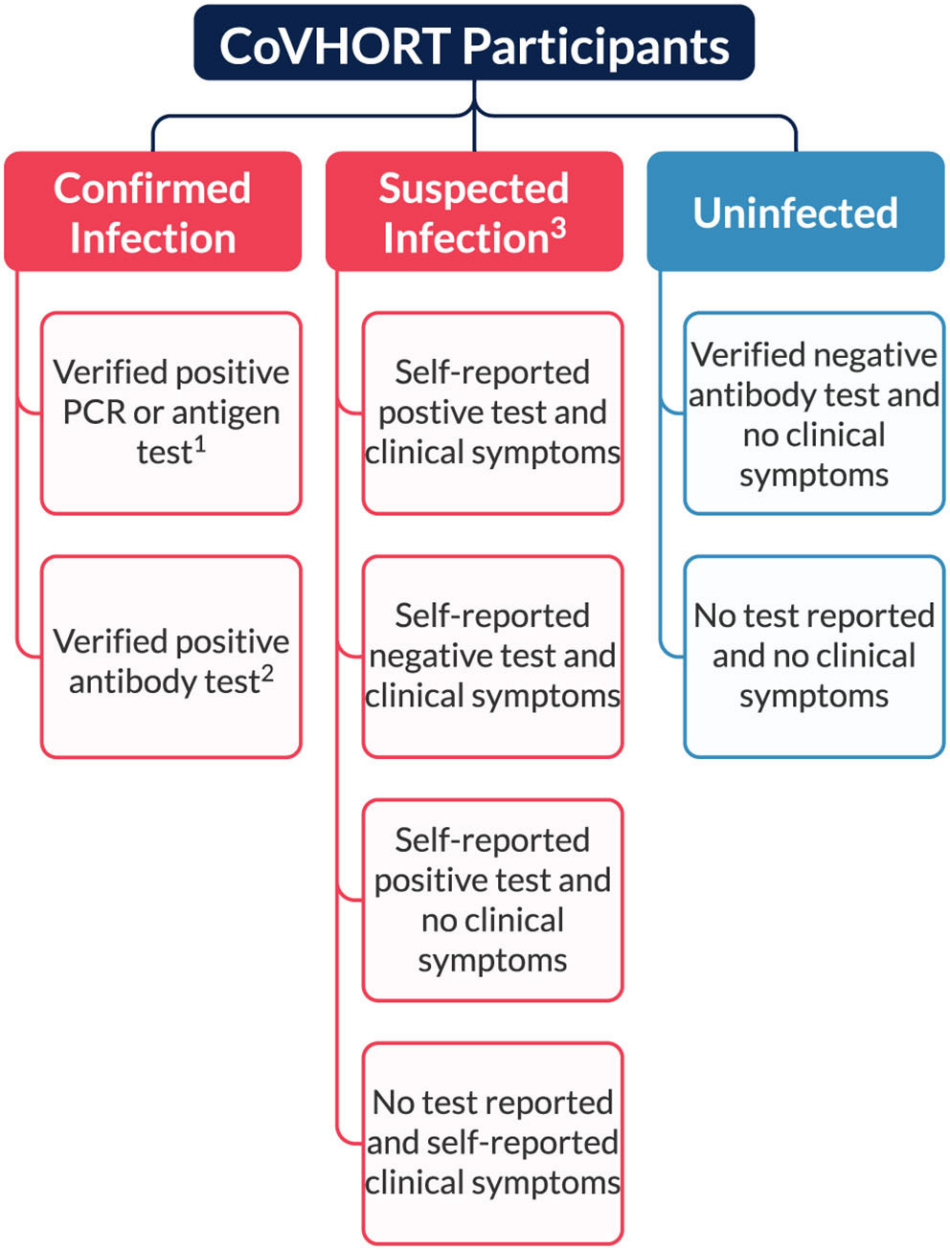
Current classification of participants based on verification of SARS-CoV-2 infection status. ^1^Verified through case investigations conducted by SAFER for state and local health departments. ^2^Verified through partnerships with the State of Arizona Antibody Testing Initiative. ^3^All self-reported test results regardless of tests (PCR, antigen, or antibody) are determined to be suspected due to inability to verify each participant's results.

Our partnerships with local health departments and collaborations with the State of Arizona COVID-19 Antibody Testing Initiative allows us to recruit residents who have laboratory confirmed current or past infections with SARS-CoV-2. Participants who are recruited from either case investigations or the antibody testing initiative are imported to our cohort with their linked laboratory test results, so we are treating these individuals as confirmed infections (Figure 2). Although other participants may report positive test results, we are not actively verifying self-reported test results with local health departments or the Arizona Department of Health Services. Thus, individuals who report a positive test result or symptoms consistent with COVID-19 illness at any point in time during follow-up are treated as suspected infections. All participants who report no symptoms of COVID-19 and/or receive a negative test result are treated as uninfected residents. While this is a limitation, we will also be able to refer participants who have not received an antibody test to the COVID-19 Antibody Testing Initiative to minimize misclassification. Additionally, at each follow-up survey we ask all uninfected participants to self-report their current SARS-CoV-2 infection status, including any COVID-like symptoms and results of any diagnostic or antibody test received since their last completed survey (see Participant Follow-up and Retention). By doing so, we are able to identify incident SARS-CoV-2 infections among our previously uninfected participants and appropriately assign surveys and survey items developed to track their illness and recovery.

#### Inclusion of Traditionally Underserved Participants

Stark differences in COVID-19 disease incidence and severity by race/ethnicity have emerged as the pandemic has progressed. Black, Indigenous and Latinx persons have higher COVID-19 infection, hospitalization, and mortality rates compared to Whites (34). These disparities arise from a constellation of social inequities that drive underlying disparities in health care access and health outcomes. This is particularly, true in tribal nations where high levels of transmission highlight the structural, social, and health inequities experienced over generations due to colonialism and institutional racism that influence susceptibility to infection and severe disease.

Arizona's diversity is a strength of this cohort, with ~30% of our residents identifying as Latinx, and 5.3% of the population is a member of one or more of the 21 independent tribal nations that fall within Arizona state boundaries. The Director of Tribal Engagement for CoVHORT (author FCM) has initiated the process to establish and develop partnerships between CoVHORT and tribal lands. While members of Tribal Nations are welcome to join the current cohort, we are not actively recruiting individuals residing on tribal lands until these relationships can be fully developed.

To develop a cohort that reflects the state's demographics, and is inclusive of Arizona's Spanish speaking population, CoVHORT investigators developed materials in both Spanish and English from the outset in collaboration with community partners. All consent forms, surveys, and recruitment materials are available in English and Spanish. Additionally, three of the five counties that the SAFER program currently conducts case investigations for are along the Arizona and Sonora, Mexico border. Latinx populations comprise 40–80% of the total population in these counties.

Participant data are regularly audited for demographic characteristics. These strategies serve as mechanisms to promote inclusion of a wide range of ages, ethnicities, countries of origin, and socioeconomic status.

#### Enrollment

Regardless of recruitment method, eligible participants are directed to the CoVHORT study website (https://covhort.arizona.edu/) whereupon they are provided an overview of the study, information about what is involved with participation, and a link to begin the informed consent process if they choose to enroll. The consent procedure lists potential risks and benefits of participation and allows participants to withdraw from the study at any time. Following completion of the consent process, participants may begin the baseline survey.

##### Current Participants

As of December 22, 2020 (day 208 of study recruitment), CoVHORT has enrolled 2,881 participants, 28.8% of our 10,000-participant goal. Of these, 16.2% have a history of COVID-19 determined by self-report of a positive diagnostic test result (Table 3). The characteristics of the participants thus far are described in Table 3. Briefly, the majority are women (65.9%), and white (87.9%), without a reported history of infection (83.8%). One in five to six, 18.0%, identify as Hispanic, Black, or Indigenous. Another 4.6% identify as multi-racial.

**Table 3:** Baseline characteristics of current participants of the Arizona CoVHORT as of December 22, 2020.

	**No. (%)**
Age (mean ± SD), y	47 ± 16
**Gender**	
Male	959 (33.3)
Female	1,898 (65.9)
Non-binary	15 (0.5)
Transgender male	3 (0.1)
Transgender female	1 (0.0)
**Race**	
AI/AN	28 (1.0)
Asian	83 (2.9)
Black	33 (1.1)
NH/PI	3 (0.1)
White	2,532 (87.9)
Mixed race	133 (4.6)
**Ethnicity**	
Hispanic	458 (15.9)
Non-hispanic	2,331 (80.9)
History of COVID-19 disease^a^	467 (16.2)

*%, percent; AI/AN, American Indian/Alaskan Native; NH/PI, Native Hawaiian or Pacific Islander; No., number; SD, standard deviation; y, years*.

a *Determined by self-report of positive PCR test for SARS-CoV-2*.

#### Participant Follow-Up and Retention

**Figure 3:**
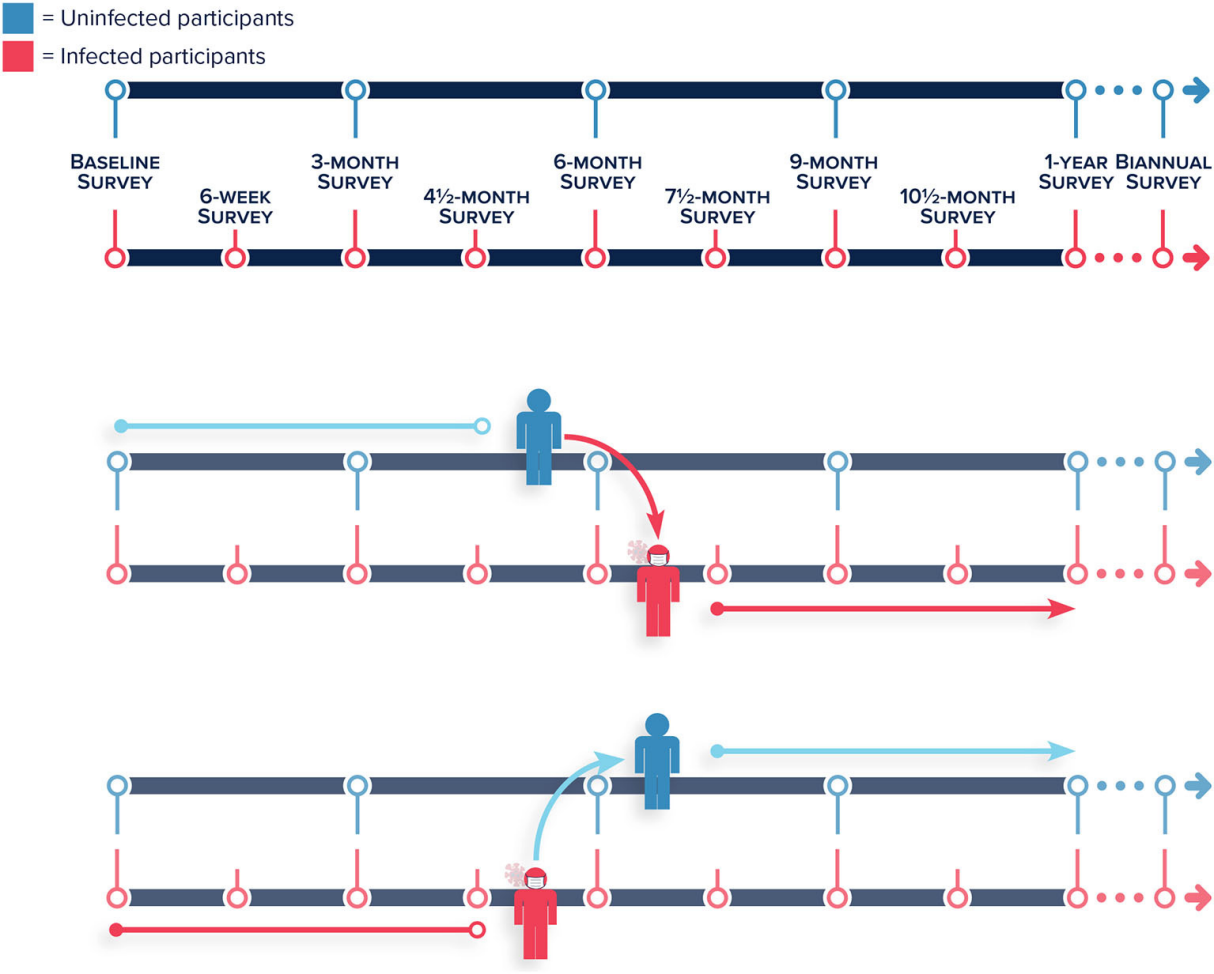
Frequency of on-cycle surveys administered to uninfected and infected participants, and the frequency of off-cycle surveys administered to only infected participants over the course of the follow-up period of the Arizona CoVHORT. (Top and Middle panel) Participants who were uninfected that self-report a COVID-19 illness or a positive test during follow up are shifted to the infected participants survey timeline including the additional “off-cycle” surveys. (Bottom panel) Infected participants that report they are no longer experiencing symptoms over the course of two “off-cycle” surveys are shifted to the uninfected participant survey timeline without the additional “off-cycle” surveys.

Planned follow up for all participants occurs at baseline, 3, 6, 9 months, and 1-year post-enrollment (Figure 3). At month 13 following enrollment, we will reduce contact to biannual for the remainder of follow-up. These “on-cycle” surveys, administered to participants with and without a prior COVID-19 diagnosis, allow us to ascertain if there is a higher prevalence of chronic conditions following infection. As one of the primary goals of the CoVHORT is to provide data regarding any long-term COVID-19 disease sequelae, we have planned additional “off-cycle” questionnaires for all participants with suspected or confirmed COVID-19 that are sent at the midpoint between each planned contact to all participants. These “off-cycle” surveys, scheduled at 1.5 months after each quarterly on-cycle survey, are used to assess acute conditions related to SARS-CoV-2 infection. All participants who are COVID-19 positive at enrollment will immediately be scheduled to complete both the on-cycle and off-cycle surveys. We will also schedule the off-cycle surveys for all participants that self-report an incident COVID-19 illness or a positive test result over the course of the follow-up period (Figure 3). These off-cycle surveys are administered as long as participants are reporting symptoms. If a participant with a confirmed or suspected SARS-CoV-2 infection reports experiencing no symptoms on two consecutive off-cycle surveys, they are redirected to only receive the on-cycle surveys for the remainder of their follow-up, or until they begin to experience symptoms again (Figure 3).

These follow-up methods also allow for the development of sub-studies of specific participants which may ask additional survey questions and/or to provide additional clinical samples. This framework allows flexibility to answer research questions pertaining to special populations, such as children, elderly, or pregnant individuals. As such, the CoVHORT will also function as a pool of Arizona residents who may be recruited for future public health outreach and research efforts conducted by the University of Arizona.

Participant retention poses a major challenge to collect follow-up data longitudinally. In an effort to address this challenge, all of our surveys are designed to take no longer than 30 min to complete and can be completed fully online. However, in the event a participant does not have access to a computer or other device with internet access or they would prefer not to complete the surveys online, we also provide the option for participants to opt in for mailed print versions of the surveys that they would mail back once completed. Understanding the importance of transparency, as well as participant interest in study progress, we are developing a page on the CoVHORT website dedicated to sharing current study descriptive data and findings with participants. We are also discussing the periodic dissemination of an electronic CoVHORT newsletter for active participants that would contain study updates and also reminders to stay active with follow-up surveys. Further, we are exploring various strategies, such as gifts or small monetary incentives, to motivate participants to remain engaged in the study.

### Data Management and Analyses

All data will be collected using Research Electronic Data Capture (REDCap), a HIPAA-compliant electronic data collection platform (35). REDCap is a secure, web-based software platform to support data capture and management in research. At the time of each follow-up questionnaire, a unique link is sent by email through REDCap to study participants. The surveys are available in English or Spanish. Additionally, participants who do not wish to complete the survey online, or are unable due to limited or restricted access to a reliable internet connection, are offered the ability to complete print versions of the surveys that can be mailed back to us in a prepaid envelope. Any differences in participants due to method of survey completion (self-administered vs. mailed print) will be evaluated and considered in the analyses. Data is stored in a password-protected database and is accessible only to the PI and paid research staff. Data management and quality control procedures are implemented by the same individuals. We understand that researchers outside of CoVHORT study team may also wish to collaborate with us or wish to have access to our participants' collected data to conduct their own analyses. We review each request on a case by case basis before granting access to our dataset. Any interested researchers should reach out directly to either of the corresponding authors to begin our internal review process.

#### Statistical Analysis

The CoVHORT is already yielding a rich dataset, and we will provide a large data repository for exploring both known and yet unknown research questions. All analyses will follow best practices in our analytical approaches and reporting, including pre-specified analysis plans, statistically defensible methods for missing data, thoughtful sensitivity analyses, and the careful use of reporting guidelines.

The primary outcome for the first aim is COVID-19 severity, categorized as severe (requiring inpatient care) or not (asymptomatic, mild, moderate). We will perform logistic regression to investigate the association of chronic health conditions (e.g., cardiometabolic conditions such as hypertension, type 2 diabetes, CVD, obesity), behavioral risk factors (mask wearing, quarantine practices, etc.) with severe COVID-19. All models will be adjusted for other known risk factors, including age, sex, race/ethnicity, and stratify analyses by sex if the interaction of comorbid condition and sex is statistically significant; we will categorize age to account for potential non-linearity. We will estimate prevalence of each comorbid condition and behavioral factor amongst persons with COVID-19, stratified by severity, age, sex and race/ethnicity. For each major symptom, symptom trajectory over time will be plotted, stratified by severity, age, sex, and race/ethnicity. With appropriate interval-censored survival analysis methods, we will estimate time to a symptom free state.

The primary outcome for the second aim is chronic health sequelae, including impaired lung function, cardiovascular disease, or neurological symptoms. We will model chronic health sequelae using generalized estimating equations (GEE) and compare the odds of chronic health sequelae between individuals with COVID-19 and those without. We will use a binomial distribution, logit link, independent working correlation structure and robust errors. GEE accounts for the longitudinal design and allows for time-varying covariates, including the crossover from COVID-19 negative to positive, while accounting for correlation of repeated measures within subjects. To reduce the effects of confounding we will use propensity score methods. Specifically, the probability of having COVID-19 will be estimated in our sample, and then we will use this propensity score in inverse probability weighting models (36). We will estimate prevalence of each of the comorbid condition amongst persons with COVID-19, stratified by severity, age, sex and race/ethnicity. We estimate that our CoVHORT will consist of 1,200 COVID-19 cases and 8,800 non-cases based on current estimates of positivity inthe state.

We will undertake sensitivity analyses for each of our primary outcomes. To account for missing data, we will use multiple imputation with chained equations; in other words, imputation models that include variables associated with missingness, the outcome, and from the analytic model. Other sensitivity analyses may center around changing definitions of cases and/or symptoms.

#### Power and Sample Size

For aim 1, we assumed that 12% of our sample of 10,000 would be positive for COVID-19, so that the number of COVID-19 positive cases will be 1,200. We further assumed that 15% of our positive sample will develop severe COVID-19 and used a type I error rate of 0.05. This sample size gives us between 80 and 90% power to detect an odds ratio between 1.55 and 1.60 for severe COVID-19, assuming a comorbid condition rate of 30 to 50% in the reference group (non-severe COVID-19 individuals) at the baseline assessment. For comorbid conditions with lower rates, such as 10%, we will have 80% power to detect an odds ratio of 1.9.

Assuming a 30% attrition and a 1% rate of non-COVID-19 participants developing a comorbid condition during follow-up, there is over 80% power to detect an odds ratio of 2.5 (aim 2). A 5% rate in the non-cases will give more than 90% power to detect odds ratios >2. We believe that the effect sizes on which we have powered the study are both feasible, based on previous research (37), and important from a public health standpoint.

## Discussion

The ongoing Arizona CoVHORT study, with 2,881 participants already enrolled, will further the understanding of the acute and chronic effects of SARS-CoV-2 infection in a diverse Southwestern U.S. population. Our prospective approach is ideal for investigating the outcomes of infection and disease course. The data we collect will allow us to identify risk factors and exposures that are associated with both the acute symptomology of COVID-19 and the development of post-COVID-19 health conditions. This study will provide an understanding of the yet uncharacterized chronic sequelae following recovery from COVID-19.

This study has many strengths, one of which is its collaborative population-based approach, which has yet to be implemented for an infectious disease other than coccidioidomycosis (Valley Fever) in the state of Arizona. Our approach includes various recruitment methods in place involving collaborations with local health departments, multiple Arizona universities, and investigators from other COVID-19-related research studies. In addition to the multiple modalities for participation, we have the means to develop a cohort that is representative of the demographic profile of the state of Arizona. This will ultimately lead to more generalizable data and conclusions regarding the causes and consequences of COVID-19. These partnerships and collaborations also allow us to recruit from a population of AZ residents with confirmed COVID-19 by conducting case investigations for state and local health departments. Our research team is comprised of transdisciplinary researchers with a range of expertise. That ensures that the tools we use are appropriate and that we have connections across populations. Furthermore, the participant study schedule and data collected from this cohort form a rich source of recruitment for sub-studies to address more focused research questions pertaining to specific topics or special populations, such as children, the elderly, or pregnant women.

Due to the survey-based nature of our study design, we acknowledge the limitations to data collection in some of the topic areas included in our surveys, including any biases introduced by self-reported data. We utilize validated questionnaires and scales throughout the survey to reduce these biases. Participant retention for the full 2 years of follow-up remains a major potential limitation. However, we are implanting strategies to retain participants for the duration of the study that include texting and email notifications to participants informing them of their next soon arriving questionnaire, reminders if questionnaires are not returned or entered within specified time periods, identification of alternate e-mails, small gift incentives to thank them for their participation, as well as letters conveying our appreciation and the importance of the research. Finally, to address any potential limitations on diversity of the cohort population, we are implementing specific recruitment efforts with tribal nations and Latinx populations of Arizona.

## Ethics Statement

This study involving human participants was reviewed and approved by the Institutional Review Board of the University of Arizona Human Subjects Protection Program. Written informed consent to participate in this study was provided by the participants or the participants' legal guardian/next of kin.

## Author Contributions

KP-B, LF, MJ, MB, RH, ZC, YK, KE, EJ, and EA conceptualized the study and developed the initial study protocol. KH, CC, EA, AS, SK, JRH, and KP-B participated in the design of the protocol. All authors critically reviewed the draft of the manuscript and approved the final version.

## Conflict of Interest

The authors declare that the research was conducted in the absence of any commercial or financial relationships that could be construed as a potential conflict of interest.
